# Supplementary material for: Effects of Palm Oil Deodorizer Distillate on the Ruminal Environment of Sheep
Source: Animals (Basel). 2024 Apr 24;14(9):1269. doi: 10.3390/ani14091269 (PMC11083554; doi:10.3390/ani14091269)
Supplement: Supplementary file 1 [file animals-14-01269-s001.zip › animals-2946764-Supplementary Figures.pdf]

Effects of Palm Oil Deodorizer Distillate on the Ruminal Environment of Sheep

D. A. das Graças[a,1], E. de Morais[b], A. C. Sodré Lima[c], S. M. de Souza[d], L. F. Sousa[e], D. C. Franco[f], A. L. da Costa da Silva[a], A. G. Maciel e Silva[d]

[a] *Laboratório de Genômica e Bioinformática, Universidade Federal do Pará, Instituto de Ciências Biológicas, Centro de Genômica e Biologia de Sistemas, Belém, Pará, Brasil.*

[b] *Instituto Federal de Ciência e Tecnologia do Pará, Castanhal, Pará, Brazil.*

[c] Instituto Federal de Ciência e Tecnologia do Amapá, Campus Porto Grande, Macapá, AP, Brazil.

[d] *Universidade Federal do Pará, Instituto de Medicina Veterinária, Castanhal, Pará, Brazil.*

[e] *Universidade Federal do Tocantins, Campus de Araguaína, Escola de Medicina Veterinária e Zootecnia.*

[f] *Universidade de São Paulo, Instituto Oceanográfico, Laboratório de Ecologia Microbiana, São Paulo, Brazil.*

[1] *Present address: Laboratório de Genômica e Bioinformática, Universidade Federal do Pará, Instituto de Ciências Biológicas, Centro de Genômica e Biologia de Sistemas, Belém, Pará, Brazil*

Corresponding author: Diego Assis. Email: diego.a87@gmail.com

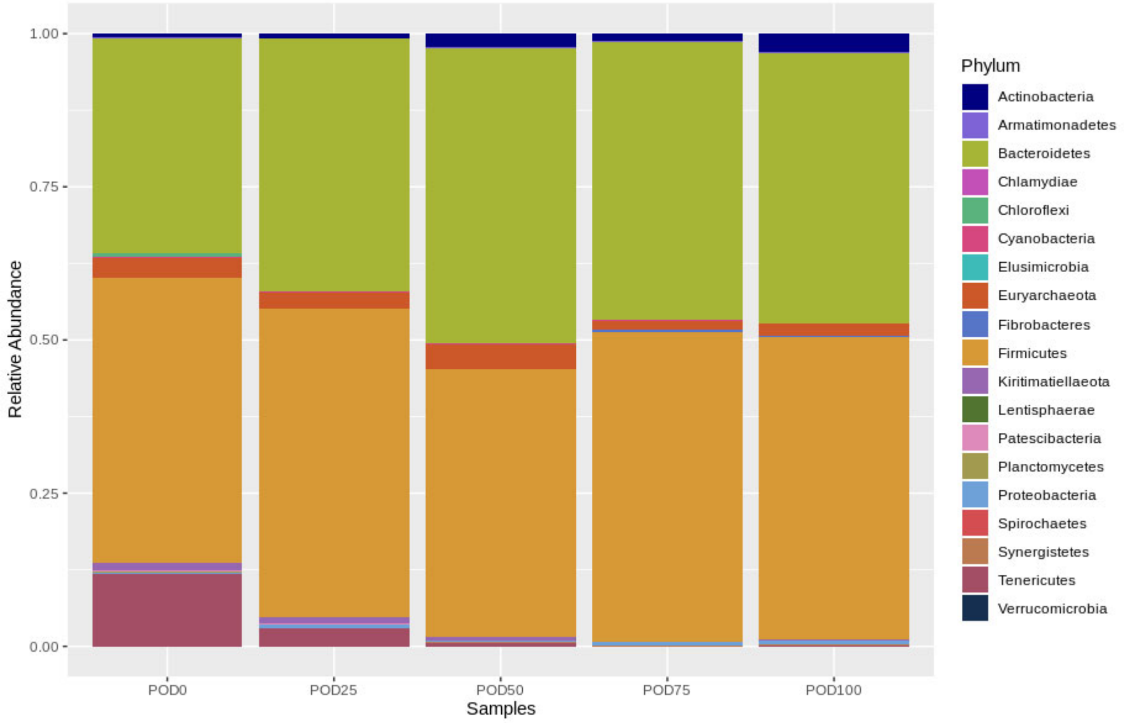

Supplementary Figure S1. Taxonomic diversity of the five samples. POD0, POD25, POD50, POD75 and POD100 correspond respectively to the inclusion of 0.0, 25.0, 50.0 75.0 and 100.0 g of POD per kg of DM
