# Supplementary material for: Effects of Palm Oil Deodorizer Distillate on the Ruminal Environment of Sheep
Source: Animals (Basel). 2024 Apr 24;14(9):1269. doi: 10.3390/ani14091269 (PMC11083554; doi:10.3390/ani14091269)
Supplement: Supplementary file 1 [file animals-14-01269-s001.zip › animals-2946764-Supplementary Tables.pdf]

Effects of Palm Oil Deodorizer Distillate on the Ruminal Environment of Sheep

D. A. das Graças[a,1], E. de Morais[b], A. C. Sodré Lima[c], S. M. de Souza[d], L. F. Sousa[e], D. C. Franco[f], A. L. da Costa da Silva[a], A. G. Maciel e Silva[d]

[a] *Laboratório de Genômica e Bioinformática, Universidade Federal do Pará, Instituto de Ciências Biológicas, Centro de Genômica e Biologia de Sistemas, Belém, Pará, Brasil.*

[b] *Instituto Federal de Ciência e Tecnologia do Pará, Castanhal, Pará, Brazil.*

[c] Instituto Federal de Ciência e Tecnologia do Amapá, Campus Porto Grande, Macapá, AP, Brazil.

[d] *Universidade Federal do Pará, Instituto de Medicina Veterinária, Castanhal, Pará, Brazil.*

[e] *Universidade Federal do Tocantins, Campus de Araguaína, Escola de Medicina Veterinária e Zootecnia.*

[f] *Universidade de São Paulo, Instituto Oceanográfico, Laboratório de Ecologia Microbiana, São Paulo, Brazil.*

[1] *Present address: Laboratório de Genômica e Bioinformática, Universidade Federal do Pará, Instituto de Ciências Biológicas, Centro de Genômica e Biologia de Sistemas, Belém, Pará, Brazil*

Corresponding author: Diego Assis. Email: diego.a87@gmail.com

Supplementary Table S1 - Ingredients and chemical compositions (g $kg^{-1}$of DM) of the experimental diets.

Ingredients		POD levels (g kg^{-1} of DM)				
		POD0	POD25	POD50	POD75	POD100
Elephant grass silage		500.0	500.0	500.0	500.0	500.0
POD		0.0	25.0	50.0	75.0	100.0
Corn		355.0	320.0	290.0	260.0	225.0
Soybean meal		125.0	135.0	140.0	145.0	155.0
Mineral and vitamin suplement[1]		20.0	20.0	20.0	20.0	20.0
Chemical composition	Silage					
Dry matter	200.8	523.5	5526.6	529.6	532.6	535.7
Organic matter	957.4	943.9	943.6	943.6	943.6	943.3
Crude protein	68.5	124.1	126.0	126.1	126.1	128.0
Ether extract	38.3	35.8	59.6	83.5	107.4	131.2
Ash	42.6	35.2	35.5	35.6	35.7	36.0
NDFap[2]	778.5	449.6	446.6	443.7	440.7	437.8
ADF[3]	489.9	269.6	269.1	268.3	267.6	267.1
Lignin[4]	77.7	-	-	-	-	-

[1]Contained per kg: 140 g of Ca, 65 g of P, 10 g of Mg, 760 mg of F, 12 g of S, 1,000 mg of Fe, 3,000 mg of Mn, 60 mg of I, 80 mg of Co and 130 g of Na; POD0, POD25, POD50, POD75 and POD100 correspond respectively to the inclusion of 0.0, 25.0, 50.0 75.0 and 100.0 g of POD per kg of DM; [2]Neutral detergent fiber corrected for ash and protein; [3] Acid detergent fiber corrected for ash and protein; [4]Lignin in sulfuric acid.

Supplementary Table S2 - Fatty acid profile of POD (g kg^{-1}).

Lauric acid (C12:0)	20.0
Myristic acid (C14:0)	15.0
Palmitic acid (C16:0)	415.0
Palmitoleic acid (C16:1)	-
Stearic acid (C18:0)	50.0
Oleic acid (C18:1)	400.0
Linoleic acid (C18:2)	100.0
Linolenic acid (C18:3)	-
Arachidonic acid (C20:0)	-
Total fatty acids	>900.0
Saturated fatty acids	504.0
Unsaturated fatty acids	496.0

Supplementary Table S3 - Regression equations of ruminal degradation parameters of dry matter (DM), organic matter (OM), crude protein and the neutral detergent fiber (NDF) of Elephant grass silage (*Pennisetum purpureum*) in diets with POD inclusion.

	Equations	PI	R^2	ED
**Dry Matter**				
POD0	$PD = 10.87+62.41(1-e^{0.0111 \times t})$	aAB	97.4	33.15
POD25	$PD = 9.86+65.77(1-e^{0.01142 \times t})$	aA	98.3	33.77
POD50	$PD = 10.93+60.34(1-e^{0.00986 \times t})$	aC	97.1	30.86
POD75	$PD = 9.96+59.65(1-e^{0.01169 \times t})$	aB	98.8	31.96
POD100	$PD = 10.24+51.47(1-e^{0.01114 \times t})$	aD	97.6	28.65
**Organic Matter**				
POD0	$PD = 10.70+75.37(1-e^{0.008 \times t})$	aAB	97.10	32.23
POD25	$PD = 9.63+77.08(1-e^{0.00862 \times t})$	aA	97.50	32.85
POD50	$PD = 10.19+69.25(1-e^{0.0075 \times t})$	aC	96.30	29.07
POD75	$PD = 9.89+70.29(1-e^{0.00866 \times t})$	aB	98.10	31.13
POD100	$PD = 9.82+54.26(1-e^{0.0099 \times t})$	aC	94.40	27.79
**Crude Protein**				
POD0	$PD = 45.84+30.64(1-e^{0.04052 \times t})$	aA	84.70	66.36
POD25	$PD = 44.32+30.75(1-e^{0.0527 \times t})$	aA	88.60	66.61
POD50	$PD = 43.42+26.85(1-e^{0.08043 \times t})$	cC	86.50	64.92
POD75	$PD = 40.22+39.41(1-e^{0.07063 \times t})$	aA	83.60	70.94
POD100	$PD = 36.66+38.21(1-e^{0.0708 \times t})$	bB	89.00	66.46
**Neutral Detergente Fiber**				
POD0	$PD = 6.18+70.00(1-e^{0.00551 \times t})$	aA	94.90	21.29
POD25	$PD = 5.64+70.13(1-e^{0.00597 \times t})$	aA	94.60	21.76
POD50	$PD = 4.48+68.22(1-e^{0.00519 \times t})$	aC	92.70	18.54
POD75	$PD = 5.71+69.38(1-e^{0.00524 \times t})$	aB	94.50	20.11
POD100	$PD = 5.96+75.27(1-e^{0.00402 \times t})$	aC	90.80	18.55

POD0, POD25, POD50, POD75 and POD100 correspond respectively to the inclusion of 0.0, 25.0, 50.0 75.0 and 100.0 g of POD per kg of DM; $PD = a + b \times (1 - e^{-ct})$. PD, potential degradability at time t (hours) of rumen incubation; a, rapidly soluble fraction; b, potentially degradable fraction; c, rate of degradation of fraction b; t, incubation time (hours). P - Parallelism; I – Identity; Equations following by equal lowercase letters in the column are parallel by the curve parallelism test; Equations following by equal capital letters in the column are identical by the curve identity test 5% error probability. ED, effective degradability.

Supplementary Table S4 - Ruminal parameters of sheep fed experimental diet.

Parameters	POD levels (g kg^{-1} of DM)					SEM	*P-value*		
	0	25	50	75	100		D	T	D x T
[1]NH_3-N (mg dL^{-1})	15.88	12.39	14.49	14.26	14.30	0.52	0.689	<0.001	0.465
[2]pH	6.68^b	6.76^b	6.67^b	7.01^1	6.98^a	0.03	<0.001	0.049	0.512
[3] MBR (min)	3.31^c	3.77^b	4.11^b	4.11^b	4.62^a	0.08	<0.001	<0.001	0.616
Protozoa (x 10^5/mL)	12.58^a	12.25^a	2.87^b	4.87^b	3.5^b	132,313.73	<0.001	-	-

NH_3-N - ammonia nitrogen; MBR, methylene blue reduction time; D - Diet; T time; D x T - Diet x time interaction; SEM, standard error of the mean, Equal letters do not differ by the SNK test.
